# A case report of lineage switch from T-cell acute leukemia to B-cell acute leukemia

**DOI:** 10.1097/MD.0000000000022490

**Published:** 2020-10-30

**Authors:** Yejing Zhu, Hui Liu, Shuna Zhang, Yanyan Liang, Meng Xiao, Yunliang Hao, Yun Guan

**Affiliations:** a Clinical college of Jining Medical University; b Jining NO. 1 People's Hospital; c Affiliated Hospital of Shandong University of Traditional Chinese Medicine, China.

**Keywords:** B cell acute lymphoblastic leukemia, lineage switch, T cell acute lymphoblastic leukemia

## Abstract

**Rationale:**

ALL is the most common form of leukemia (75% to 80%), it is characterized by clonal expansion of the lymphoid blasts in bone marrow, blood, and other tissues, which can be divided into T lineage and B lineage. Although relapse of acute leukemia is common, a change of immunophenotype at relapse only occurs rarely. Some of these cases have been labeled “lineage switch”.

**Patient concerns:**

A 31-year-old man had multiple lymph nodes in the neck, and the lymph nodes on the right side adhered to the surrounding tissues. His lymphocytes ratio in blood was up to 86.3%. Flow cytometry of the bone marrow aspirate showed positive results for CD2, CD5, CD7, cCD3, TDT, CD4, CD8, and CD10, negative results for CD34, CD117, CD33, HLA-DR, CD19, and CD20. Twenty six months later, the patient felt pain in the neck and shoulder after touching. His lymphocytes of blood were 109.9×109 /L. 43 fusion genes and positive BCR/ABL was detected. Flow cytometry of the bone marrow aspirate showed pro B lymphocytes accounted for 85.54%, and positive expression of CD38, CD10, CD34, CD33, TDT, CD9, and HLA-DR. Moreover, the RT-PCR data showed the patient expressed high level of T cell and B cell development transcription factors.

**Diagnoses:**

Upon examination, the patient was initially diagnosed with T-lineage pro cell ALL. BM morphologic analysis presented complete remission (CR) after systemic chemotherapy. Twenty six months later, we discovered the patient was diagnosed with B-lineage acute lymphocytic leukemia.

**Interventions:**

Systemic chemotherapy is first given when a patient was diagnosed with T-cell acute lymphoblastic leukemia. After the patient happened linage switch, we adjusted the treatment plan, and the patient was complete remission after 1 course of treatment.

**Outcomes:**

Our case provides information of lineage switch from T-ALL to B-ALL in this report, which is never seen in our knowledge.

**Lessons:**

This lineage switch from T-ALL to B-ALL is never reported beforemoreover, the RT-PCR data showed the patient expressed high level of T cell and B cell development transcription factors. Its early recognition can let doctor provides appropriate therapy to patient.

## 1 Introduction

Acute lymphoblastic leukemia (ALL) is characterized by clonal expansion of the lymphoid blasts in bone marrow, blood, and other tissues, which can be divided into T lineage and B lineage. T-cell acute lymphoblastic leukemia (T-ALL) and B-cell acute lymphoblastic leukemia (B-ALL) are both aggressive malignant neoplasm of the bone marrow and blood. B-ALL is the most common type of acute lymphoblastic leukemia, while T-ALL only accounts for approximately 20% of all cases of ALL. ALL is more frequently seen in adults than in children, although the incidence declines with age.^[1]^ The clinical presentations include hyperleukocytosis in extramedullary lymph nodes and other organs, frequent central nervous system infiltration and mediastinal mass in the thymus.^[2]^

"Lineage switch” describes the condition where acute leukemia converts to a different lineage at relapse compared with that during the initial treatment based on the standard French-American-British (FAB) criteria, which can be manifested as the transformation of cell morphology, histochemical and immune types. Lineage switch can be regarded as a phenomenon of a variety of biphenotypic or bilineal leukemia. Moreover, it may be part of the biologic process of mixed-lineage leukemia in certain cases.^[3,4]^ Lineage switch is bidirectional, the rate of conversion from ALL to acute myelocytic leukemia (AML) was reported to be 6% to 9%, most of which occurred in children.^[5,6]^ It is also possible that AML may convert to ALL, which, however, is extremely rare, and the prognosis can be variable.^[7]^ In this case, we reported a rare case of linage switch, which should be the first reported case of a lineage switch from acute T cell leukemia to B cell leukemia to our knowledge.

## 2 Case presentation

**Figure 1:**
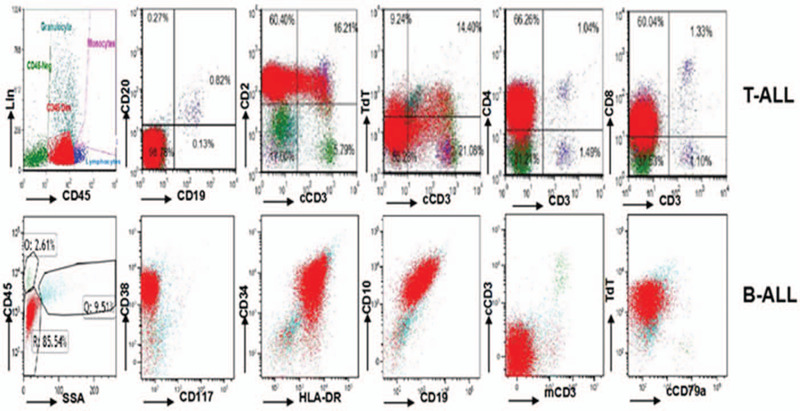
Flow cytometry of the bone marrow aspirate of the patient, including he was in T-ALL and B-ALL period.

A 31-year-old man was diagnosed with T-lineage pro cell ALL in October 2014, he had no family genetic history. At first, the patient had multiple lymph nodes in the neck, and the lymph nodes on the right side adhered to the surrounding tissues. The white blood cell count (WBC) was 65.46 × 109/L, with a neutrophil ratio of 6.6% and a lymphocyte ratio of 86.3%. The immature cells accounted for 85% of the peripheral blood cells. Bone marrow smear suggested 98% primary and juvenile cells, and lymphoblastic leukemia (L2) was considered. No fusion gene was detected (Table 1). Flow cytometry of the bone marrow aspirate showed positive results for CD2, CD5, CD7, cCD3, TDT, CD4, CD8, and CD10, negative results for CD34, CD117, CD33, HLA-DR, CD19, and CD20. The patient was diagnosed with T-lineage acute lymphocytic leukemia (Table 2 and Fig. 1) and the diagnosis was very clear. There were no diagnostic challenges during our therepy we have given Cyclophosphamide (CTX) and VDLD [vindesine sulfate (VDS), Dexamethasone (DXMS), Pegaspargase, Demethoxydaunor Ubicin] chemotherapy after excluding chemotherapy contraindication. We strictly followed the chemotherapy regimen and it was useful, but we ignored the individual difference of patient, the patients immunity function was very low and he was developed a lung infection. After a course of treatment, the patients BM morphologic analysis showed he was complete remission (CR).

**Table 1:**
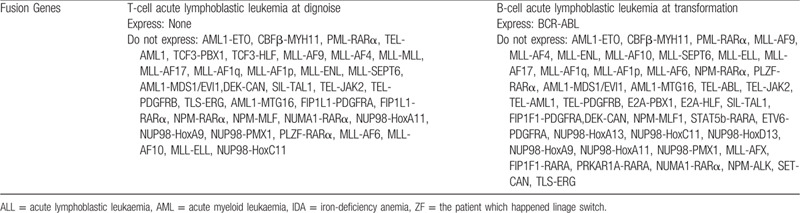
Differences between T-cell acute lymphoblastic leukemia and B-cell acute lymphoblastic leukemia in fusion genes.

**Table 2:**
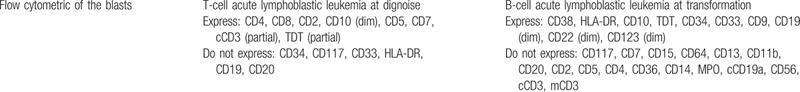
Differences between T-cell acute lymphoblastic leukemia and B-cell acute lymphoblastic leukemia in flow cytometry.

Twenty six months later, the patient felt pain in the neck and shoulder after touching. His white blood cell count (WBC) was 136.22 × 10^9^/L and lymphocyte was 109.9 × 10^9^/L. The BM cell morphology suggested 94% native lymphocytes 43 fusion genes and positive BCR/ABL was detected. The quantification of BCR/ABL P210 was 134.2967%, which was higher than the international standard of 120.8670% (Table 1). Flow cytometry of the bone marrow aspirate showed pro B lymphocytes accounted for 85.54%, and positive expression of CD38, CD10, CD34, CD33, TDT, CD9, and HLA-DR, while weak signals of CD19, CD22, and CD123 were detected (Table 2, Fig. 1). Therefore, the patient was diagnosed with B-lineage acute lymphocytic leukemia, and the diagnosis was very clear. According to the WHO diagnosis and classification, He belonged to the poor prognosis group. In strict accordance with the guidelines for the diagnosis and treatment of adult acute lymphoblastic leukemia (2018 edition), the patient was treated with induction, maintenance and consolidation programs. By evaluating the related results of the patients blood and bone marrow, the patient was complete remission after chemotherapy. There were no diagnostic challenges during our therepy.

**Figure 2:**
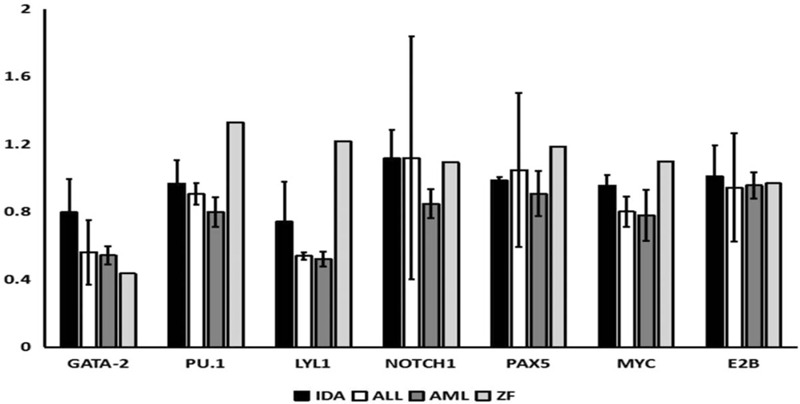
The expression of GATA-2, PU.1, LYL1, NOTCH1, PAX5, MYC, and E2B in different hematological diseases was analyzed by qPCR.

To further understand the mechanism of linage switch, several transcription factors involved in the development of hematological diseases were examined, including GATA-2, PU.1, LYL1, NOTCH1, PAX5, MYC ,and E2B. GATA-2 and PU.1 regulate the differentiation of stem cells to progenitor cells, and LYL1 and NOTCH1 can promote the differentiation of lymphoid progenitors to T-lineage cell, while PAX5, MYC, and E2B regulate the B lineage cell development. The peripheral blood samples of patients with iron deficiency anemia, acute myeloid leukemia and acute lymphoid leukemia were collected, and the expression levels of the above genes were analyzed by RT-PCR, the results of which were compared with those obtained from the patient (Fig. 2). Compared with the patients with other hematological diseases, the patient had higher levels of LYL1, NOTCH1, PAX5, MYC, and E2B, all of which contribute to the development of T and B cells.

## 3 Discussion

Although the mechanism of lineage switch in acute leukemia remains unclear, several hypotheses have been proposed to explain the phenomenon, including the involvement of bipotential progenitor cells, cell reprogramming and dedifferentiation, clonal selection and seeding of donor cells, all of which are proposed to participate in the conversion of leukemic cell lineage. Besides, the microenvironment may influence all proposed mechanisms by modulating the genome plasticity of the cells and change the leukemia outcome at relapse.^[8–10]^

We found that the most discussed are myeloid/lymphoid progenitor cell hypothesis and chemotherapy related hypotheses. According to the common myeloid/lymphoid progenitor cell hypothesis, uncommitted progenitor cells that have both early myeloid and T- or B-lymphoid markers lose 1 lineage marker during the maturation process and differentiate toward the other lineage. According to the primary chemotherapy related hypotheses, chemotherapy might suppress or eradicate the leukemic clone apparent at diagnosis, thereby permitting the expansion of a subclone with a different phenotype; alternatively, chemotherapy could modify the original leukemic clone by amplifying or suppressing the normal differentiation programs, thus causing a shift in the expression of the phenotypic features. In some cases, topoisomerase II inhibitors (epipodophyllotoxins and anthracyclines) affect secondary AML. Secondary ALL, however, is very rare and is associated with only a few fusion genes, such as IgA and T-cell receptor gene rearrangements. In this case, we found that the patient had early myeloid and T- or B-lymphoid markers, and the patient also had undergone a series of chemotherapy.^[5,9]^ Although we did not find specific evidence, the above-mentioned effects may not be excluded.

We reported a case of lineage switch from T-ALL to B-ALL in this case report. Changes were observed in both histochemistry and immunotyping, which met the diagnostic criteria for lineage switch. During the initial treatment for T-ALL, expressions of CD4, CD8, CD5, CD7, cCD3, and partial TDT were detected, and lack of mature B cells and myeloid antigen hematopoietic precursor cells was observed. At recurrence, positive expressions of BCR-ABL, CD8, HLA-DR, CD10, TDT, CD34, CD33, CD9, and dim CD19 were detected and the markers of T-ALL was lost to be detected. It is also one of the most common changes that increase or loss of phenotypic markers in linage switch. Moreover, several transcription factors which were related to cell development were also examined, and the results suggested that the expression levels of both T cell and B cell development transcription factors were higher in the patient, compared with other cases with iron-deficiency anemia, acute myeloid leukemia and acute lymphoid leukemia, most of which only one kind of transcription factors was up-regulated. The laboratory results may provide additional insights in the linage switch from T-ALL to B-ALL. Lineage switch may exists various forms and its frequency is probably underestimated, its early recognition can let doctor provides appropriate therapy to patient.

## Acknowledgments

We are very grateful to the teachers of Jining NO.1 People's Hospital and Jining Medical College for their support and Mr. Zhou Fu's cooperation.

## Author contributions

**Data curation:** Yejing Zhu, Hui Liu, Yun Guan.

**Funding acquisition:** Yun Liang Hao.

**Project administration:** Meng Xiao, Yun Liang Hao.

**Resources:** Hui Liu, Shuna Zhang, Yan Yan Liang.

**Writing – original draft:** Yejing Zhu, Hui Liu, Yun Guan.

**Writing – review & editing:** Yejing Zhu, Yun Guan.
